# A sensitive three monoclonal antibodies based automatic latex particle-enhanced turbidimetric immunoassay for Golgi protein 73 detection

**DOI:** 10.1038/srep40090

**Published:** 2017-01-05

**Authors:** Yanyan Xia, Han Shen, Yefei Zhu, Hongpan Xu, Zhiyang Li, Jin Si

**Affiliations:** 1 Department of Clinical Laboratory, the Affiliated Drum Tower Hospital of Nanjing University Medical School, Nanjing 210008, China; 2 Department of Clinical Laboratory, the Second Affiliated Hospital, Nanjing Medical University, Nanjing 210011, China; 3 State Key Laboratory of Bioelectronics, School of Biological Science and Medical Engineering, Southeast University, Nanjing 210096, China

## Abstract

Golgi protein 73 (GP73) is a novel and potential marker for diagnosing hepatocellular carcinoma (HCC) that has been found to be abnormally elevated in liver disease. A latex particle-enhanced turbidimetric immunoassay (LTIA) was recently introduced and licensed for application in a variety of automated clinical chemistry analyzers. However, no studies have reported sufficient data on analytical performance of this method when using 3 monoclonal antibodies for GP73 measurement. The experimental conditions were firstly optimized and range of linearity, diagnostic potential, clinical relevance were compared with the LTIA based on polyclonal antibodies and ELISA. Dilution tests for the LTIA using 3 monoclonal antibodies produced a calibration curve from 10 to 350 ng/mL while the polyclonal antibodies produced the curve from 20 to 320 ng/mL. The detection limit was achieved at 1.82 ng/mL concentration. Within-run CV was obtained in the range of 1.5–2.9% and ROC curves indicated sensitivity and specificity of the LTIA based on 3 monoclonal antibodies were 96.7% and 93.3%, respectively, higher than for the polyclonal antibodies (94.6% and 72.4%) and ELISA (70.0% and 83.3%). Therefore, the LTIA assay based on 3 monoclonal antibodies is thus applicable in quantification of GP73 concentration in automated biochemistry analyzers.

Hepatocellular carcinoma (HCC) is currently ranked among the most common primary malignant cancers worldwide and is the third and fifth leading cause of death from cancer globally in men and women, respectively^1^,^2^,^3^. Due to lack of effective strategies for early diagnosis and pre-clinical screening for HCC in high-risk populations, the majority of patients can be treated only with loco regional therapies, resulting in limited survival benefits and tumor recurrence in 50–80% of patients at 5 years after treatment^4^,^5^. The 5-years survival rate for patients with HCC was disappointedly low (at 14%) as compared to approximately 27% for early diagnosed patients^6^. Early detection and effective treatment are therefore crucial for improving the survival and quality of life of patients with HCC. Serum AFP is the most commonly used biomarker for HCC but the clinical diagnostic accuracy for detecting early HCC has been questioned as its sensitivity is only around 60%^7^,^8^,^9^. Besides, many individuals with HCC express only slight elevation of AFP while 80% of the smaller case (tumors <3 cm) show no elevation whatever, causing the lost of its sensitivity^10^. A novel serum biomarker that exhibits superior diagnostic accuracy is therefore required in diagnosing the HCC.

Golgi protein 73 (GP73) is also called Golgi membrane protein 1 or Golgi phosphoprotein 2^11^,^12^. It is expressed at low levels in biliary epithelial cells in healthy livers and up-regulated in the hepatocytes of patients with liver diseases^13^. It is also strongly up-regulated in hepatocytes from patients with HCC disease^14^. Accumulating evidence from studies has recently revealed that the sensitivity and specificity of GP73 for HCC diagnosis were more superior than AFP^15^,^16^,^17^. A meta-analysis compared results from GP73 assays with AFP, showing that the sensitivities in the diagnosis of HCC were 0.77 (95% CI: 0.75–0.79) and 0.62 (95% CI: 0.60–0.64) for GP73 and AFP, respectively, while their specificities were 0.91 (95% CI: 0.90–0.92) and 0.84 (95% CI: 0.83–0.85)^18^. The GP73 was therefore proposed as a novel surrogate marker for HCC diagnostics. Many analytical methods have been developed for GP73 detection, such as Western blotting (WB)^19^, double antibody sandwich ELISA^20^ and lectin capture strategies^21^. Meantime, these methods suffer from drawbacks, such as complicated manipulation procedures and long analysis time^22^. Consequently, it is imperative to develop a rapid and automated method for GP73 detection, with good sensitivity and calibrator stability.

Latex particle-enhanced turbidimetric immunoassay (LTIA) consists of latices which are coated with antibodies that react with a specific analyte that can be applied to relate the triggered particle aggregation to an analyte concentration by means of a fast and easy measurement like turbidimetry^23^,^24^,^25^,^26^. It is efficient and has high degree of automation, and is also suitable for a large clinical sample testing. Polyclonal antibodies are most commonly used for detecting the GP73, due to their concerted action that displays multiple specificities and bind to several epitopes, however, their diagnostic accuracy has been questioned, for their low specificity^27^. Monoclonal antibodies exhibit unprecedented specificity to their antigenic target but this extreme specificity may hamper the efficacy of any individual monoclonal antibody^28^,^29^. The concept on combining several monoclonal antibodies to increase their detection efficacy and overcome the limitations of having low neutralizing potency appears to be logical. We therefore designed the LTIA using latex bead-immobilized 3 GP73-monoclonal antibodies. The performance of the LTIA was evaluated on a BECKMAN AU5400 automated analyzer. The experimental conditions for this assay were optimized and linearity range and diagnostic potential compared with the LTIA based on polyclonal antibodies. We evaluated its analytical properties by comparing the results with ELISA assay in 80 HCC patients and 80 healthy individuals.

## Results

### Characteristics of anti-GP73 antibodies

**Figure 1:**
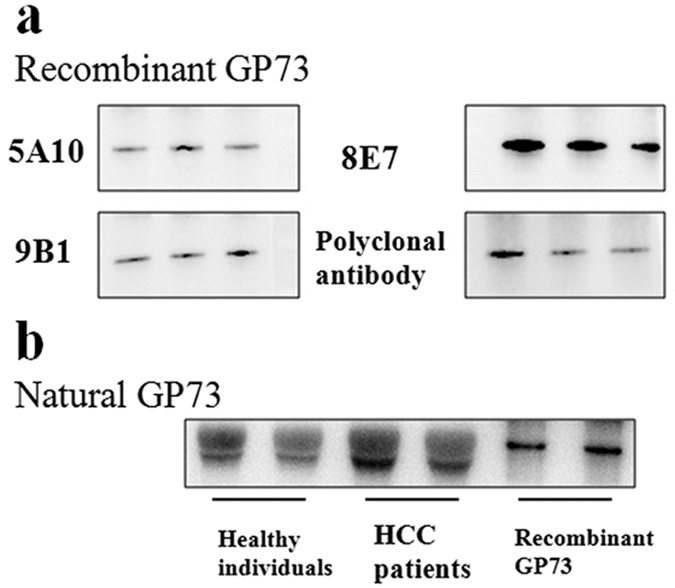
Characteristics of anti-GP73 antibodies. The specificity of anti-GP73 antibodies were determined by WB analysis with recombinant GP73 (**a**) and native GP73 (**b**).

Characterization of anti-GP73 antibodies was carried out to determine their clinical application value and make their full use in disease diagnostics. WB enables the detection of interest proteins using primary antibodies. In this experiment, the recombinant GP73 protein was perfectly recognized by 5A10, 8E7, 9B1 and polyclonal antibody (Fig. 1a). To investigate whether our antibodies could recognize natural GP73, serum samples, which included healthy individuals, HCC patients and recombinant GP73, were subjected to WB assay using 5A10. The recombinant GP73 comprised of full-length GP73 sequences and His-label, which was consistent with results that the molecular weight was slightly larger than the natural GP73 (Fig. 1b). Moreover, the results also showed specific reaction between the 5A10 and natural GP73, and higher expression level of GP73 in the HCC patients than in healthy individuals (Fig. 1b) (8E7, 9B1 and polyclonal antibody had the same results as in the WB assays, details not shown). The full-length gels were showed in Supplementary Fig. S1. Those results revealed that the specificity of anti-GP73 antibodies to GP73 protein was successfully obtained.

### Method optimization

**Figure 2:**
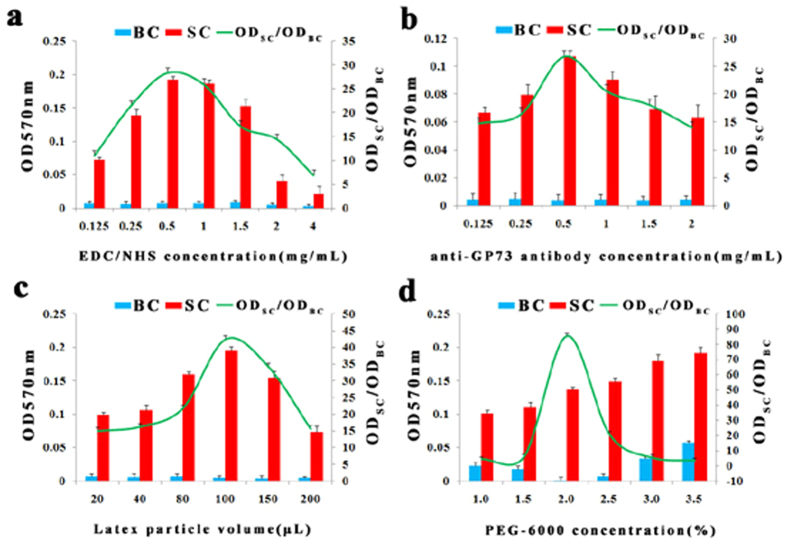
Method optimization of LTIA based on 3 monoclonal antibodies. The major variables were; (**a**) concentration of EDC and NHS, (**b**) amount of antibodies, (**c**) amount of latex-immobilized antibody reagent and (**d**) concentration of PEG-6000. The recombinant GP73 (200 ng/mL) and PBS solution acted as standard control (SC) and blank control (BC) respectively.

All work was undertaken to optimize assay performance within the constraints of the BECKMAN analyzer and with existing reagents available for the analyzer. The screen presentation of data provided absorbance plots with change of optical density (OD) against the concentration of variable for several combinations of the samples. The recombinant GP73 (200 ng/mL) and phosphate buffer (PBS) solution acted as standard control (SC) and blank control (BC) respectively. Our objective was to choose conditions in which the OD_SC_/OD_BC_ absorbance was higher. The results from the LTIA based on 3 monoclonal antibodies are illustrated in simplified form in Fig. 2. The OD value increased with addition of 1-ethyl-3-[3-dimethylaminopropyl carbodiimide hydrochloride (EDC)/N-hydroxysulfosuccinimide (NHS) concentration and anti-GP73 antibody concentration in the proper range. Excessive addition of EDC/NHS and anti-GP73 antibody decreased the surface charge densities caused by the huge steric hindrance, leading to the decreased OD value^30^ (Fig. 2a and b). The latex particle density in the assay solution increased with increasing volume of latex particle. Ideally, a neutral density particle that is equal to that of the buffer is required when the particle is to be suspended. If the particle has a significantly higher density than the diluents, then the OD value may be decreased^31^ (Fig. 2c). A proper PEG concentration had major influence on both the sensitivity and assay range parameters and the OD value increased with increased PEG concentration in the range of 1.0–2.0%. However, the excessively increased PEG concentration was accompanied by decreased OD value, owing to the steric exclusion of the tethered PEG chains^32^ (Fig. 2d). We finally added 0.5 mg/mL of EDC and NHS with 0.5 mg/mL 3 anti-GP73 monoclonal antibodies to activate latex. 2% of polyethylene glycol (PEG-6000) was selected in the final protocol and 100 μL of antibody-immobilized latex beads were added in to the cuvette to start the turbidimetric immunoreactions. Turbidity was monitored at 570 nm. Similarly, the LTIA based on polyclonal antibodies was optimized and the final optimal parameter results were; 0.5 mg/mL of EDC and NHS with 2.0 mg/mL anti-GP73 polyclonal antibodies were used to activate the latex. 2% of PEG-6000 and 100 μL of antibody-immobilized latex beads were then used to start the turbidimetric immunoreaction and turbidity was monitored at 570 nm. The finally chosen parameters after linear and co-optimization were as follows.

### Linearity

**Figure 3:**
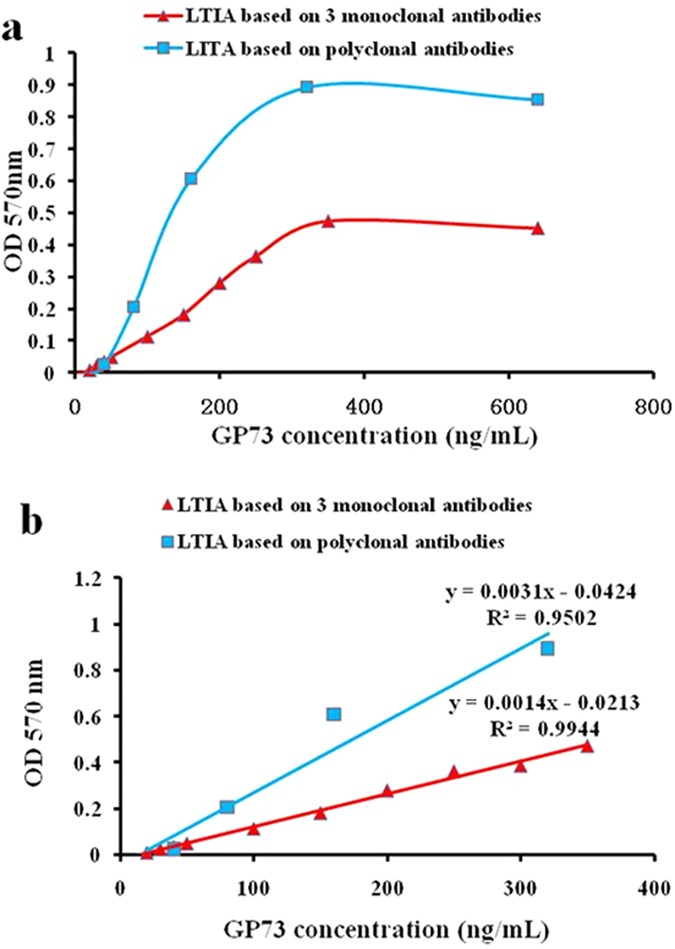
Linearity of LTIA. Linearity tests for the GP73 were performed using LTIA with 3 monoclonal antibodies and polyclonal antibodies on a BECKMAN AU5400 analyzer, respectively. The standard curve was established using GP73 standard samples concentration from 1.5 to 640 ng/mL (**a**). The figure shows a very good linear response in the concentration range from 10–350 ng/mL for the 3 monoclonal antibodies and 20–320 ng/mL for the polyclonal antibodies, respectively (**b**).

All the parameters were optimized and a series of GP73 standard samples concentrations from 1.5 to 640 ng/mL were used to assess the calibration curve. The measurements at each dilution were taken in triplicate at different times. The standard curve was mapped with the expected value of GP73 as the X-axis and the measured OD_570_ as the Y-axis. Although the OD value increased with the increasing concentration of GP73 in a certain range, the results had “hook effect” when the concentration of the GP73 was increased, with the curve showing declining trend (Fig. 3a). For the LTIA based on 3 monoclonal antibodies, the deviations from theoretical values did not exceed 5% while within the measuring range of 10–350 ng/mL, indicating no lack of parallelism and showing good linearity. The linear regression equation was y = 0.0014x − 0.0213, R^2^ = 0.9944 (Fig. 3b). For the LTIA based on polyclonal antibodies, the deviations from theoretical values did not exceed 5% within the measuring range of 20–320 ng/mL, indicating no lack of parallelism and showing good linearity. The linear regression equation was y = 0.0031x − 0.0424, R^2^ = 0.9502 (Fig. 3b). These results showed a very good linear response in the concentration range from 10–350 ng/mL for the 3 monoclonal antibodies and 20–320 ng/mL for the polyclonal antibodies, respectively. Considering the linear correlation, the R^2^ value for the LTIA based on 3 monoclonal antibodies (0.9944) was much better than the LTIA based on polyclonal antibodies (0.9502). Therefore, the good linear correlation for the LTIA based on 3 monoclonal antibodies is conducive for the future experiments.

### Precision

To determine the precision for the LTIA, we performed a replication study. Three pools of serum containing GP73 with different concentrations were prepared, aliquoted, and stored at −20 °C, to provide the quality-control materials for this study. Within-run coefficient of variation (CV) was then assessed by running 20 replicates of each sample pool randomized in a single analysis. Day-to-day total CV was analyzed in duplicate in two runs per day with a single calibration for 20 days. As shown in Table 1, the within-run CV was 1.514–2.901%, total CV was 3.632–7.234% in LTIA based on 3 monoclonal antibodies. On the other hand, the within-run CV in LTIA based on polyclonal antibodies was 1.940–3.636 and total CV was 7.103–9.659 (Supplementary Table S1). Batch CV was assessed by running monoclonal antibodies from three different production batches for each sample pool. Because of the heterogeneity for the monoclonal antibodies in different batches, it was necessary to reestablish the standard curve for each batch. The results showed that the batch CV was 2.032–10.149 (Supplementary Table S2).

### Lower detection limit

The analytical detection limit was estimated as analyte concentration that can be discriminated with >99% confidence from zero calibrator^26^. It was calculated by the mean absorbance value plus three standard deviations for 10 replicates from the zero calibrator. The detection limit for the LTIA based on 3 monoclonal antibodies was 1.82 ng/mL, with 1.67 mean and 3 SD of 0.15. LTIA based on polyclonal antibodies was 2.11 ng/mL, with mean of 1.97 and 3 SD of 0.14. These results showed slightly better detection limit for the LTIA based on 3 monoclonal antibodies than polyclonal antibodies.

### Carry-over

We measured the PBS solution before and after the measurement of the sample with high concentration of GP73 (500 ng/mL). The mean percentage deviation was compared to the Acceptable Change Limit (ACL) according to the ISO 5725-6 standard. The ACL for interpreting the measured difference was based on the analytical imprecision (CVa) using the formula ACL = 2.77 CVa. The CVa is the mean CV obtained from the precision experiment^24^. The mean value within-run CV for the LTIA based on monoclonal antibodies and polyclonal antibodies were 2.43% and 2.98%, respectively. We concluded that a mean percentage deviation greater than 2.77 CVa (6.73% and 8.26%) represented a probable difference in analyte concentration. The mean CV of LTIA based on monoclonal antibodies and polyclonal antibodies for three PBS samples were 3.85% and 4.46%, these results showing variations within the accepted limits. The results indicated that there was no detectable sign of carry-over of GP73 on a BECKMAN AU5400.

### Stability

Stability of this assay was assessed by measuring the GP73 concentration of serum standards with 3 monoclonal and polyclonal antibody-immobilized latex beads on the preparation day and after storage in dilute form at 4 °C for three, six and nine days. Five serum samples covering a wide range of GP73 concentrations (12.5–200 ng/mL) were used for the stability study. Fresh serum samples were collected from patients and aliquoted into polypropylene tubes and each aliquot was used only once. The CV of the LTIA based on monoclonal antibodies for each standard was <6.73% (Supplementary Fig. S2). Similarly, the CV of the LTIA based on polyclonal antibodies was <8.26%. The results indicated that there were no significant variations in the absorbance of these standards during the assessment period.

### Interference

**Figure 4:**
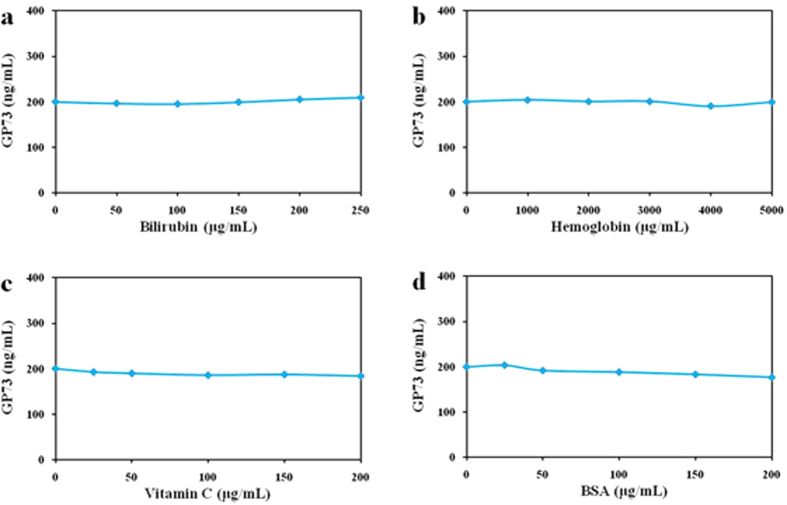
Interference tests of LTIA based on 3 monoclonal antibodies. The CV of GP73 was within 6.73% of the original concentration.

Interference tests were performed by adding potentially interfering substances to serum samples and examining the changes in absorbance values. In LTIA based on 3 monoclonal antibodies, we assessed the possibility that high concentrations of bilirubin might interfere with the immunoreactions by supplementing five bilirubin samples with concentration up to 250 μg/mL. The CV was calculated at 2.73% and result showed variation within the accepted limits (6.73%) as seen in Fig. 4a. Similarly, concentration of hemoglobin (up to 5000 μg/mL) and vitamin C (up to 200 μg/mL) did not alter assay precision, as the CVs were 2.31% and 3.12%, respectively (Fig. 4b and c). This is because bovine serum albumin (BSA) is present in many commercial calibrator and quality control materials. We also checked the effect of cross-reaction from the BSA and results showed that the CV of the BSA (up to 200 μg/mL) was 4.38%, it did not affect assay precision (Fig. 4d). Similarly, the CV of LTIA based on polyclonal antibodies was <8.26% (Supplementary Fig. S3). These results indicated that the substances tested here did not interfere with the ability of the assay to accurately measure the GP73.

### Serum GP73 concentrations in clinical samples

**Figure 5:**
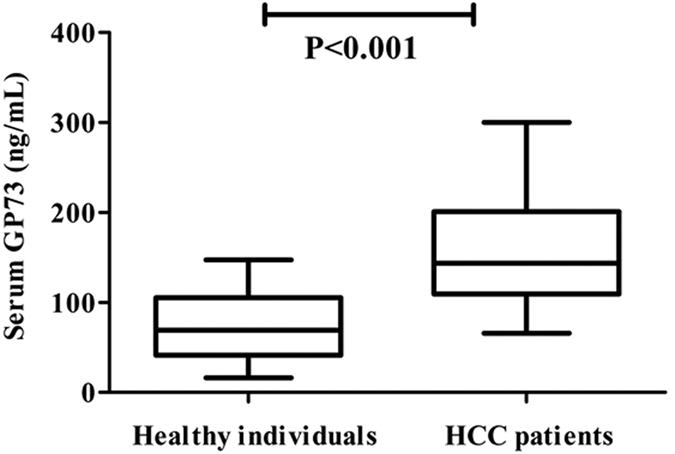
Serum GP73 concentrations in clinical samples. GP73 concentration in HCC patients and healthy individuals. The statistical difference between 80 HCC patients and 80 healthy individuals was significant (*P* < 0.001).

To validate the use of the GP73 in clinical serum samples, we examined the GP73 levels in 80 HCC patients and 80 healthy individuals. The results suggested that the LTIA based on 3 monoclonal antibodies had been successfully developed for the quantification of serum GP73. The expression concentration of the GP73 detected by the LTIA ranged from 16.27 to 147.19 ng/mL in healthy subjects, with a median of 69.23 ng/mL and mean of 77.02 ng/mL (95% IC: 68.27–85.78 ng/mL). On the other hand, the expression concentration of GP73 detected by the LTIA ranged from 65.73 to 300.02 ng/mL in the serum from the HCC, with a median of 143.68 ng/mL and mean of 155.35 ng/mL (95% IC: 143.12–167.58 ng/mL). The statistical difference between the HCC patients and healthy individuals was significant (*P* < 0.001, Fig. 5).

### Correlation between LTIA and ELISA

**Figure 6:**
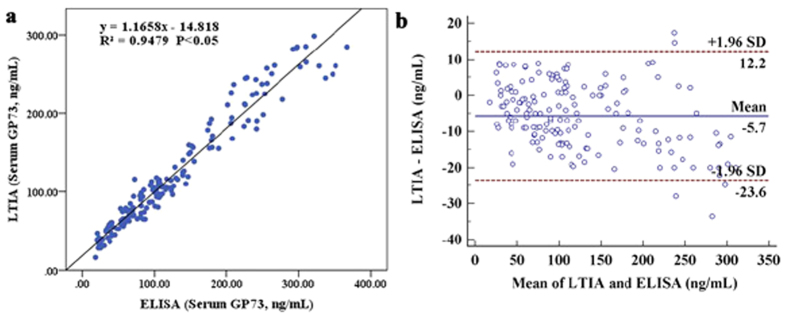
Correlation between LTIA and ELISA for detecting GP73. (**a**) The figure shows the results from regression analysis of measured serum GP73 concentrations between LTIA (y-axis) and ELISA (x). (**b**) Bland–Altman plots showing the difference between the LTIA and ELISA readings at different ambient LPL levels. The x-axis shows the means of 2 readings and y-axis shows the difference between the two methods. The dashed line represents the 95% confidence limits for the differences. A regression line shown on the graph demonstrated that the mean difference between the methods was small and that there was no consistent bias preference between the methods.

To investigate the differences between LTIA and ELISA methods in detecting the serum GP73 and preliminary clinical application of LTIA, regression statistics were analyzed. As shown in Fig. 6a, regression statistics calculated in the tested serums (n = 160) gave y_(LTIA)_ = 1.1658x_(ELISA)_ − 14.818, R^2^ = 0.9479, indicating a good correlation between the two methods. A Bland-Altman plot was constructed to visualize the differences between the two methods^33^. The difference plot confirmed that there was a difference between the values measured with LTIA and ELISA, and the 95% confidence limits of the differences were ±17.9 ng/mL (Fig. 6b). The mean difference between the two immunoassays was −5.7 ng/mL, suggesting that there was no consistent bias between the two methods. A somewhat greater variance was observed at a GP73 concentration >230 ng/mL. This was not considered to reduce the reliability or potential usefulness of the assay, since the potential interest regarding risk assessment lies in the low range.

### ROC Curves

**Figure 7:**
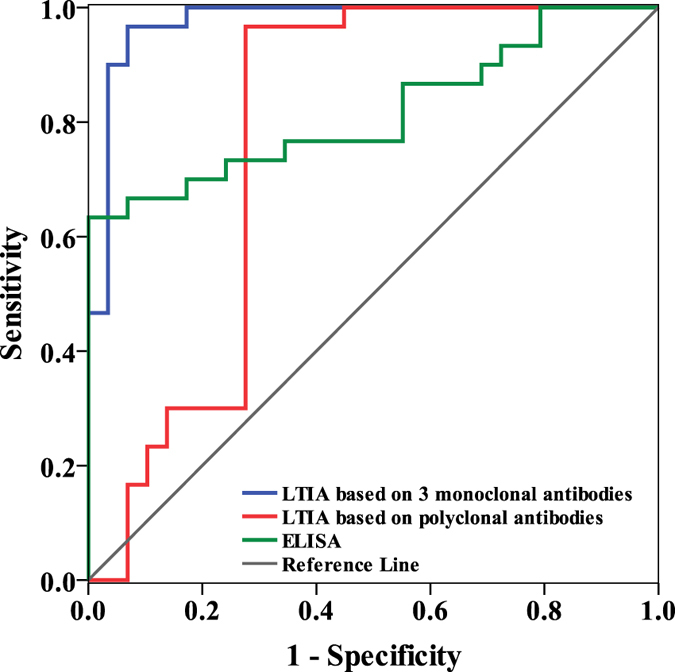
Receiver operating characteristic (ROC) curve analysis. The areas under the ROC curve for LTIA based on 3 monoclonal antibodies, polyclonal antibodies and ELISA were 0.976, 0.774 and 0.820, respectively.

To evaluate the diagnostic potential for the LTIA based on 3 monoclonal antibodies in detecting the GP73 HCC biomarker, ROC curve analysis was performed (Fig. 7). ROC analysis and area under ROC curve (AUROC) were calculated to evaluate the sensitivity, specificity and accuracy of the LTIA based on 3 monoclonal antibodies, polyclonal antibodies and ELISA in detecting HCC. The AUROC for the LTIA assay based on 3 monoclonal antibodies was 0.976 (95% CI: 0.938~0.999), with a sensitivity of 96.7% and specificity of 93.3% at the optimal cut-off point of 109.6 ng/mL. The AUROC for the LTIA assay based on polyclonal antibodies was 0.774 (95% CI: 0.638~0.910), with a sensitivity of 94.6% and specificity of 72.4% at the optimal cut-off point of 119.3 ng/mL. The AUROC for ELISA was 0.820 (95% CI: 0.710~0.930), with a sensitivity of 70.0% and specificity of 83.3% at the optimal cut-off point of 113.4 ng/mL. The ROC curve analysis demonstrated that the LTIA based on 3 monoclonal antibodies performed much better than the other two assays in differentiating the HCC from non-HCC patient. In addition, the sensitivity and specificity for the LTIA based on 3 monoclonal antibodies was higher than in other studies^34^,^35^. The LTIA method based on 3 monoclonal antibodies can therefore be satisfactorily applied in the clinical detection of serum GP73.

## Discussion

HCC is a major public health concern worldwide^36^. Early detection of HCC is critical for accurate treatment and improvement of health and survival of patients^37^,^38^. Even though the AFP is a specific serum marker for HCC, it is of minimal use as a diagnostic tool because its low sensitivity and specificity^39^,^40^. GP73 is proving to be a better biomarker for the HCC detection when compared to other reported markers, and it is possible to be utilized in the detection of early stages of HCC progression^41^. In particular, it was observed that the expression of GP73 was significantly associated with tumor grade and invasiveness, such as tumor number, presence of vascular invasion, advanced stage, deteriorated liver function and poor performance status^17^,^42^,^43^, indicating that the GP73 would also be a potential predictor for HCC prognosis. It is meaningful to therefore measure the expression levels of GP73 by using developed novel ultrasensitive quantitative immunological detection assays.

In order to determine the GP73 concentration in ordinary clinical laboratories, we in this study developed and tested a LTIA based on 3 monoclonal antibodies for measurement of GP73 concentration, in both HCC patients and healthy individuals. Polyclonal antibodies were most commonly used in previously reported protocols for the LTIA. However, compared with polyclonal antibodies, the monoclonal antibodies can be produced unlimitedly as characteristics for mono-antibody from the same fusion cell were the same compared to polyclonal anti-bodies from different immunized animals which make it difficult to standardize the measurement. The monoclonal antibodies that exhibit extreme specificity may also hamper the efficacy of any individual monoclonal antibody. The concept of combining several monoclonal antibodies to increase their detection efficacy and overcome the limitations for low neutralizing potency appears to be logical. These 3 monoclonal antibodies were selected due to the 3 monoclonal antibodies paired with each other respectively in ELISA assay, indicating that they can recognize different site for the same antigen and more easily facilitate the aggregation of the latex-immobilized antibody reagent. The present method has presented use of 3 monoclonal antibodies with higher sensitivity and specificity (96.7% and 93.3%). This novel detection method took great advantage over the polyclonal antibodies (94.6% and 72.4%) and ELISA (70.0% and 83.3%). Measurement using the ELISA requires pretreatments of samples, including heating and pre-dilution, and also requires 3 h to complete the measurement of GP73. The novel assay presented herein does not require any pretreatment of samples and takes only 10 min in an automated analyzer. Within-run CV obtained in this study was in the range of 1.5–2.9%. This superior reproducibility from our results adds further value to this convenient assay system. As the 3 monoclonal antibodies recognized distinct epitopes and paired with each other, the analytical detection limit for our assay was 1.82 ng/mL, making it suitable for detection of HCC.

The reproducibility, simplicity and full automation with widely used clinical chemistry analyzer, are key to routine clinical diagnosis. Our assay based on 3 monoclonal antibodies is applicable in quantifying the concentration of GP73 on automated biochemistry analyzers and offers the advantages of being precise, easy to perform, more rapid with higher sensitivity and specificity than the other mentioned tests. This study confirmed that the detection method based on LTIA is a sensitive, specific and easily implemented tool for measuring the GP73 concentrations. This new detection method therefore holds great potential for development and use in the clinical laboratory.

## Methods

### Reagents

Polystyrene latex particles (JSR Corporation, Tokyo, Japan), BSA (ProLiant Biologicals, USA). NHS and EDC were purchased from Yake Corporation (Suzhou, China). PEG-6000 was from Biosharp. Interfering reagents containing hemoglobin, bilirubin, and vitamin C were from JSYH Corporation (Beijing, China). All chemicals used in this experiment were of analytical reagent grade. Deionized water was used throughout the study.

### Serum samples

To determine the reference range of this assay, a total of 160 serum samples from Drum Tower Hospital of Nanjing Medical University were used to determine GP73, including 80 patients with HCC (52 males and 28 females, mean age was 58 years old) and 80 healthy individuals (49 males and 31 females, mean age was 51). All serum samples were collected using standard protocol and stored at −80 °C until use. The study was carried out in accordance with the Code of Ethics of the World Medical Association (Declaration of Helsinki) for experiments involving humans. The study was approved by Ethics Committee of Nanjing Medical University. An educated and written informed consent was obtained from all the subjects before obtaining the blood samples.

### Preparation of anti-GP73 monoclonal antibodies and polyclonal antibodies

3 anti-GP73 monoclonal antibodies can paired with each other respectively in ELISA assay (5A10, 8E7 and 9B1) were produced by intraperitoneally injecting female BALB/C mouse with hybridoma against human recombinant GP73^20^. We generated the polyclonal antibodies by immunizing rabbits with purified recombinant GP73. The monoclonal antibodies from the mouse ascitic fluid and polyclonal antibodies from the rabbit serum were purified with protein-A column (GE Healthcare) and then desalted with HiTrap Desalting column (GE Healthcare). The concentration of the antibodies was determined by the OD. All animal experiments described in this work were approved by the Institutional Ethical Committee of Animal Experimentation of Nanjing Medical University. The experiments were carried out strictly in accordance with governmental and international guidelines on animal experimentation. All efforts were made to minimize the usage amount of animals and the suffering during experiments according to the request of Biosafety and Animal Ethics.

### WB analysis

The WB analysis was applied to validate the specific reaction for the purified 3 monoclonal antibodies and polyclonal antibody to recombinant GP73 and natural GP73. The recombinant GP73 was extracted from HepG2 cells, while the natural GP73 was from serum samples, including healthy individuals and HCC patients. The recombinant and natural GP73 proteins were separated by sodium dodecyl sulfate-polyacrylamide gel electrophoresis. Briefly; 10 ng of GP73 protein extract was resolved on a sodium dodecyl sulfate-12% polyacrylamide gel and transferred onto nitrocellulose membranes for detection. The blots were incubated with three anti-GP73 monoclonal antibodies (diluted 1:5000) and polyclonal antibody (diluted 1:1000), followed by the addition of HRP conjugated goat anti-mouse (1:5000) and goat anti-rabbit secondary antibody (1:1000), respectively. Specificity of the anti-GP73 antibodies were confirmed by WB analysis with specific GP73 peptide. The peroxidase activity was detected by the enhanced chemiluminescence system.

### Protocol for latex-immobilized antibody reagent

To investigate the optimal experiment performance for LTIA, an optimization of the assay reagent was performed. The major variables were concentration of EDC and NHS, amount of antibodies, amount of latex-immobilized antibody reagent, concentration of PEG-6000, and absorbing wavelength. Polystyrene latex particles with a diameter of 107 nm (100 μL with 5% wt/vol) were suspended in 50 mM HEPES buffer (pH 7.0) and then incubated with 3 monoclonal antibodies in a shaker. After 30 min incubation at room temperature, EDC and NHS with the same concentration were added for 30 min, followed by the addition of 1% BSA. After 30 min, the antibody-immobilized latex beads were resuspended in 1.0 mM Tris-HCl (pH 6.8) containing 1% BSA and then ultrasonicated until they became translucent. The reagent for the immobilized latex beads was stored at 4 °C and used for measurement of GP73.

### Preparation of calibrators

Standards were prepared with the recombinant GP73 in PBS (pH 7.4) generated by BL21 cells in our laboratory. In-house calibrators for GP73 measurement were prepared with the Standards. To determine the values for the in-house calibrator, calibrators with known concentrations were used for comparison.

### Assay procedure

Sample processing, pipetting steps and measurements were performed automatically on a BECKMAN AU5400 automated analyzer. Briefly, 10 μL of samples and 240 μL of reagent 1 (10 mM Tris-HCl (pH 7.0), 150 mM NaCl, 0.01% Tween-20 and PEG-6000) were injected into the reaction cuvette. After 5 min of incubation, reagent 2 (antibody-immobilized latex bead) was added into the cuvette to start the turbidimetric immunoreaction. After another 5 min, the GP73 concentration was calculated from the difference in the absorbance values between the 5^th^ and 10^th^ time points, with 570 nm wavelength. The calibration curve was obtained using a series of working GP73 calibrators and used to calculate the values of the serum samples.

### Statistical analysis

Statistical analysis was performed using SPSS 19.0. Differentiation of serum GP73 concentrations between the two groups (healthy controls and HCC group) were compared according to a two-tailed Student’s t-test. Pearson’s correlation coefficient was calculated between LTIA and ELISA. ROC curve analysis was performed to evaluate the diagnostic potential for the LTIA compared to ELISA, to identify the optimal cut-off values, sensitivity and specificity. *P* < 0.05 was considered statistically significant.

## Additional Information

**How to cite this article**: Xia, Y. *et al*. A sensitive three monoclonal antibodies based automatic latex particle-enhanced turbidimetric immunoassay for Golgi protein 73 detection. *Sci. Rep.* **7**, 40090; doi: 10.1038/srep40090 (2017).

**Publisher's note:** Springer Nature remains neutral with regard to jurisdictional claims in published maps and institutional affiliations.

## Supplementary Material

Supplementary Information

## Figures and Tables

**Table 1:** Precision data for LTIA based on 3 monoclonal antibodies.

Concentration (ng/mL)	Within-run (n = 20)			Total (n = 20)		
	Mean	S.D.	CV (%)	Mean	S.D.	CV (%)
25	25.421	0.733	2.885	25.215	1.824	7.234
100	100.671	1.524	1.514	100.721	6.539	4.507
150	152.029	4.41	2.901	151.989	5.520	3.632
