# Supplementary material for: A sensitive three monoclonal antibodies based automatic latex particle-enhanced turbidimetric immunoassay for Golgi protein 73 detection
Source: Sci Rep. 2017 Jan 5;7:40090. doi: 10.1038/srep40090 (PMC5215377; doi:10.1038/srep40090)
Supplement: Supplementary Information [file srep40090-s1.pdf]

# Supplementary Information

## A sensitive three monoclonal antibodies based automatic latex particle-enhanced turbidimetric immunoassay for Golgi protein 73 detection

Yanyan Xia[1], Han Shen[1], Yefei Zhu[2], Hongpan Xu[2], Zhiyang Li[1, 3*], and Jin Si[1*]

1. Department of Clinical Laboratory, the Affiliated Drum Tower Hospital of Nanjing University Medical School, Nanjing 210008, China.

2. Department of Laboratory Medicine, the Second Affiliated Hospital, Nanjing Medical University, Nanjing 210011, China.

3. State Key Laboratory of Bioelectronics, School of Biological Science and Medical Engineering, Southeast University, Nanjing 210096, China.

*Corresponding author: Zhiyang Li, Department of Clinical Laboratory, the Affiliated Drum Tower Hospital of Nanjing University Medical School, Nanjing 210008, China. Email: 29313783@qq.com

Jin Si, Department of Clinical Laboratory, the Affiliated Drum Tower Hospital of Nanjing University Medical School, Nanjing 210008, China. Email: sijin_efy@njmu.edu.cn

This supplementary file contains Table S1, S2, Fig. S1-3 and their legend.

**Table S1. Precision data for LTIA based on polyclonal antibodies**

| Concentration (ng/ml) | Within-run (n=20) | | | Total (n=20) | | |
|---|---|---|---|---|---|---|
| | Mean | S.D. | CV (%) | Mean | S.D. | CV (%) |
| 25 | 25.327 | 0.853 | 3.368 | 26.483 | 2.558 | 9.659 |
| 100 | 103.801 | 2.013 | 1.940 | 102.641 | 7.291 | 7.103 |
| 150 | 158.44 | 5.761 | 3.636 | 153.924 | 12.642 | 8.213 |

**Table S2. Batch CV for LTIA based on 3 monoclonal antibodies**

| Concentration (ng/ml) | Batch CV | | |
|---|---|---|---|
| | Mean | S.D. | CV(%) |
| 25 | 26.500 | 2.347 | 8.857 |
| 100 | 109.661 | 11.129 | 10.149 |
| 150 | 153.070 | 3.111 | 2.032 |

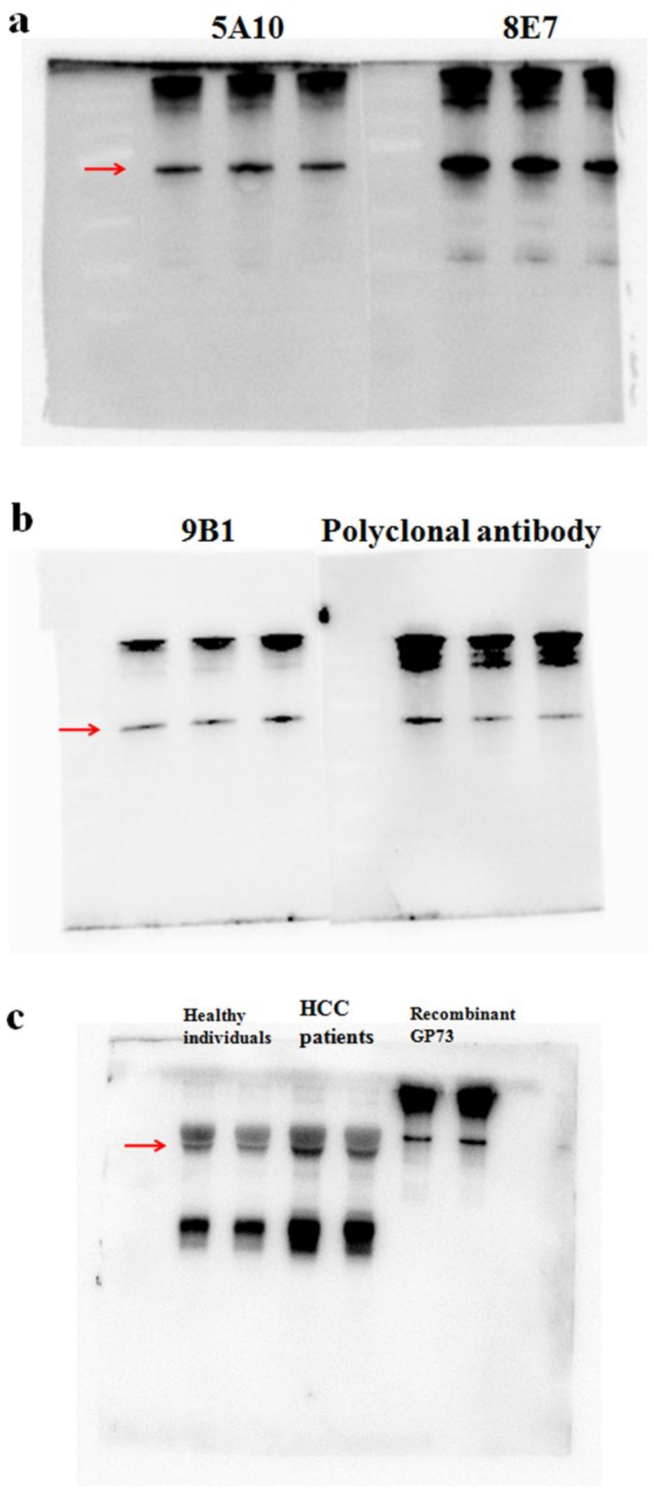


**Fig. S1. Characteristics of anti-GP73 antibodies.** The full-length gels were showed. The specificity of anti-GP73 antibodies were determined by WB analysis with recombinant GP73 (a and b) and native GP73 (c). The red arrow indicated the target band.

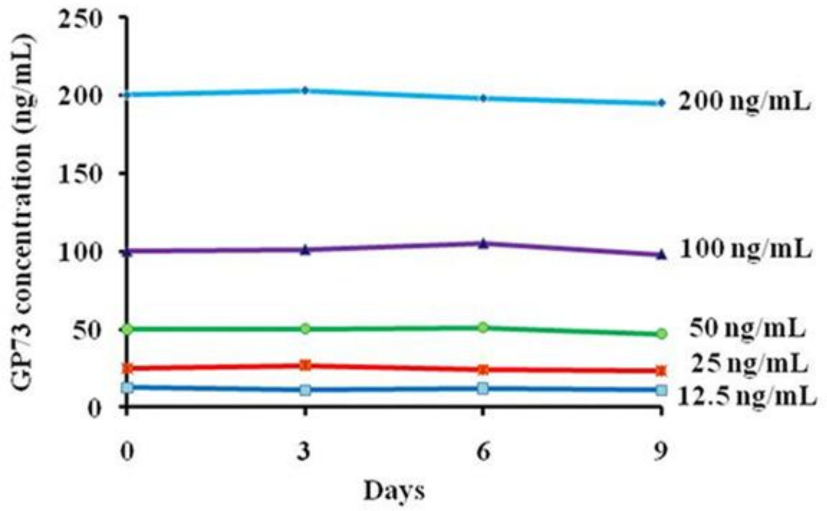


**Fig. S2. The stability of antibody-immobilized latex beads.** Absorbance measurement of serum standards with antibody-immobilized latex beads stored diluted at 4 ℃ for nine days.

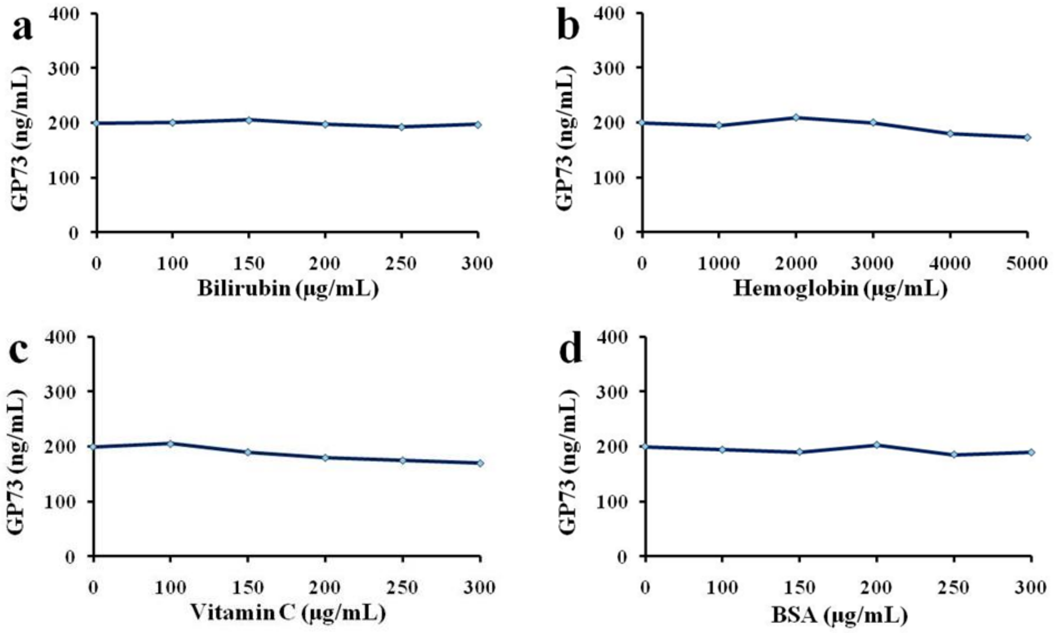


**Fig. S3. Interference tests of LTIA based on polyclonal antibodies.** The CV of GP73 was within 8.26% of the original concentration.
